# Characterization of complete mitochondrial genome of the Brown Accentor *Prunella fulvescens* on the Tibet Plateau

**DOI:** 10.1080/23802359.2017.1357436

**Published:** 2017-07-26

**Authors:** Zhen Cao, Guopan Li, Cai Chen, Shaobin Li

**Affiliations:** a College of Life Science, Yangtze University, Jingzhou, Hubei, China;; b Institute of biological medicine, Yangtze University, Jingzhou, Hubei, China

**Keywords:** Accentor, mitochondrial genome, passerine, *Prunella*, Tibet Plateau

## Abstract

The Brown Accentor (*Prunella fulvescens*) is a poorly known bird species distributed in Eurasia high-montane areas. In this paper, we described the whole mitochondrial genome of the Brown Accentor. The entire mitochondrial sequences were determined using long-range PCR and conserved primer walking approaches. The results demonstrated that the whole mitochondrial genome of *P. fulvescens* was 16,837 bp in length with 54.1% A + T content; the genome harboured the same gene order as that of other passerine birds, including 13 protein-coding genes, 2 rRNA genes, 22 tRNA genes and 1 non-coding control region. The control region (D-loop) of *P. fulvescens* was located between tRNA-Gln and tRNA-Phe with 1247 bp length. These mitochondrial data are potentially important for the further studies on molecular evolution and conservation genetics in avian species.

The Brown Accentor (*Prunella fulvescens*) is a small oscine passerine of the Old World. This species is widespread in Eurasian high-montane areas, with its breeding regions cross the Himalayas, Tibetan Plateau and Central Asia and the altitudinal distributions ranging from 1000 m to more than 4000 m above sea level (Zheng 2005; Lu 2006; Hatchwell 2017). Currently, similar to other accentor species except Dunnock (*P. modularis*; Burke et al. 1989) and Alpine Accentor (*P. collaris*; Davies et al. 1996), the Brown Accentor is still poorly understood and the knowledge on their natural history is mainly based on species accounts (Hatchwell 2017) and simple descriptions by Lu (2006). In this study, we described the whole mitochondrial genome of *Prunella fulvescens* based on next-generation sequencing. The newly sequenced complete mitochondrial genome sequence will provide basic data for further molecular phylogenetic studies on this poorly known species.

Blood sample of the Brown Accentor was collected by puncturing the brachial vein from an female individual (id pf_1707) caught by mist nets, on 15 July 2016 at Tianjun County (37°18' N, 99° 01' E; 3416 m a.s.l.), northeastern of Tibet Plateau. The sample was quickly stored in TES buffer (50 mM EDTA, 1% SDS, 50 mM Tris). Genomic DNA was extracted from blood samples according to the protocol of TIANamp Genomic DNA kits (Tiangen, Beijing). The blood sample (id pf_1707) is stored in the Room 420 of College of Life Science at Yangtze University. The complete sequence of the Brown Accentor mitochondrial genome was determined by long-range PCR and conserved primer walking approaches.

The results revealed that the entire mitochondrial genome of the Brown Accentor comprised 16,837 bp nucleotides in length, which exhibited the typical mitochondrial structure of passerine birds, including 13 protein-coding genes, 2 rRNA genes, 22 tRNA genes and a non-coding control region. The overall nucleotide composition includes A (30.2%), T (23.9%), G (14.9%) and C (31.0%), with a total A and T content of 54.1%. The entire mitochondrial sequence has been deposited in GenBank with accession number of KY471556.

Among the 37 genes, 28 were encoded on heavy strand and the remaining genes on the light strand. All protein-coding genes of the *Prunella fulvescens* mitochondrial genome started with ATG codon, except for COI and ND2 with GTG. For terminate codon usage, most of the genes terminate with TAA or TAG, and ND5 terminated with AGA, ND1 and COXI with AGG, and the COIII and ND4 genes had an incomplete termination codon T. The control region of *P. fulvescens* was located between tRNA-Gln and tRNA-Phe with 1247 bp length, which contained several conserved sequences involved in the replication and transcription of mitochondrial genome.

Phylogenetic analyses were conducted with mitochondrial data of this study and 11 other bird species from the GenBank database. The topology of the tree was inferred using maximum-likelihood analyses in the program MEGA7 (Kumar et al. 2016). Execution model was statistically well supported by high bootstrap values at most nodes (Figure 1). The phylogenetic tree revealed that three *Prunella* species clustered in a clade and were closely related with Estrildidae species, which accorded with the traditional morphology-based taxonomies and recent molecular taxonomies (Zheng 2005; Clements et al. 2016; Gill and Donsker 2017). Similar results were reached through BLAST analysis in GenBank that *P. montanella* and *P. strophiata* are the closest relatives to *P. fulvescens* with 95% and 94% sequence similarity, respectively. The complete mitochondrial genome of the Brown Accentor could provide fundamental information for further molecular phylogenetic studies on Prunellidae species.

**Figure 1.**
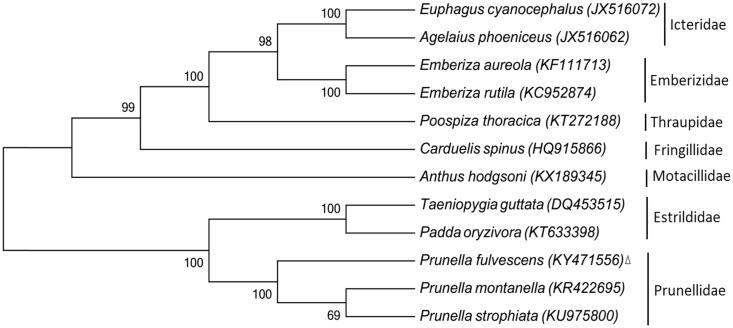
Maximum-likelihood phylogeny of 12 related bird species based on their complete mitochondrial genome (the GenBank accession number in parentheses and family names in the right). Data from this study were marked with a triangle. Numbers at branches indicate bootstrap values from 1000 replications.
